# Dopamine axons in dorsal striatum encode contralateral visual stimuli and choices

**DOI:** 10.1523/JNEUROSCI.0490-21.2021

**Published:** 2021-07-12

**Authors:** Morgane M Moss, Peter Zatka-Haas, Kenneth D Harris, Matteo Carandini, Armin Lak

**Affiliations:** 1 Department of Physiology, Anatomy and Genetics, University of Oxford, Oxford, OX1 3PT, UK; 2 UCL Institute of Ophthalmology, University College London, London WC1E 6BT, UK; 3 UCL Queen Square Institute of Neurology, University College London, London WC1E 6BT, UK

## Abstract

The striatum plays critical roles in visually-guided decision making and receives dense axonal projections from midbrain dopamine neurons. However, the roles of striatal dopamine in visual decision making are poorly understood. We trained male and female mice to perform a visual decision task with asymmetric reward payoff, and we recorded the activity of dopamine axons innervating striatum. Dopamine axons in the dorsomedial striatum (DMS) responded to contralateral visual stimuli and contralateral rewarded actions. Neural responses to contralateral stimuli could not be explained by orienting behavior such as eye movements. Moreover, these contralateral stimulus responses persisted in sessions where the animals were instructed to not move to obtain reward, further indicating that these signals are stimulus-related. Lastly, we show that DMS dopamine signals were qualitatively different from dopamine signals in the ventral striatum, which responded to both ipsi- and contralateral stimuli, conforming to canonical prediction error signaling under sensory uncertainty. Thus, during visual decisions, DMS dopamine encodes visual stimuli and rewarded actions in a lateralized fashion, and could facilitate associations between specific visual stimuli and actions.

## Introduction

Central to survival is the ability to execute appropriate actions based on incoming visual information in order to obtain rewards. Dorsal striatum plays critical roles in visually-guided decision making (Ding and Gold, 2013; Hikosaka, 2006). Previous studies have identified prominent projections from visual cortical areas to the dorsal striatum (Hintiryan et al., 2016; Hunnicutt et al., 2016; Khibnik et al., 2014), and have shown that neurons in the dorsal striatum are active during visually-guided behavior, particularly responding to contralateral visual stimuli (Hikosaka et al., 1989; Kawagoe et al., 2004; Peters et al., 2021) and reflecting visual evidence accumulation during decision making (Ding and Gold, 2010), contributing causally to visual decisions (Doi et al., 2020). In addition to cortical inputs, striatum receives dense axonal projections from midbrain dopamine neurons (Björklund and Dunnett, 2007; Haber, 2014). However, the roles of striatal dopamine in visual decision making have remained relatively unknown.

Several lines of evidence suggest that dopamine signals in the dorsal striatum play crucial roles in visual decision making. First, the activity of midbrain dopamine neurons correlates with statistical decision confidence during visual decision making (Lak et al., 2017; Lak et al., 2020). Second, dopamine depletion in dorsal striatum alters striatal sensory responses (Ketzef et al., 2017). Third, manipulation of cortico-striatal neurons, terminating in the dorsal striatum, biases choices in 2-alternative sensory decision tasks (Znamenskiy and Zador, 2013). Fourth, the strength of cortico-striatal synapses increases in a stimulus-selective manner as animals learn to perform a sensory decision task (Xiong et al., 2015) and these synapses are strongly modulated by dopamine signals innervating the dorsal striatum (Calabresi et al., 2007; Reynolds and Wickens, 2002). Therefore, striatal dopamine signals are well-placed to entrain associations between stimuli and actions during visual decisions.

We recorded the activity of dopamine axons in the striatum in mice trained to perform a visual decision task with asymmetric reward payoff. We found that dopamine axon activity in the dorsomedial striatum (DMS) encoded the contrast of contralateral visual stimuli, regardless of subsequent movement direction. In fact, the contralateral stimulus responses persisted in a task in which the stimulus instructed animals specifically not to move in order to receive the reward, indicating that these responses are truly driven by contralateral stimulus, rather than the action that follows the stimulus presentation. Additionally, we observed contralateral action-aligned signals in these DMS dopamine axons, but only in rewarded trials. For comparison, we also recorded the activity of dopamine axons in the ventral striatum (VS), which responded to both ipsi- and contralateral stimuli and trial outcomes, and conformed to canonical prediction error signaling under sensory uncertainty. These results reveal distinct roles for dopamine signals in different regions of striatum during visual decisions, and suggest that DMS dopamine signals could facilitate associations between contralateral visual stimuli and contralateral actions.

## Material and methods

### Mice and surgeries

The presented data were collected from 6 male and 3 female mice (DAT-Cre backcrossed with C57/DL6J; B6.JLSl6a3tm1.1(cre)Bkmn/J; https://www.jax.org/strain/006302) aged between 10 and 24 weeks. Mice underwent surgery during which a metal headplate was implanted, as well as either one or two optic fibers following viral injection. Mice were anaesthetized with isoflurane (induction: 3% in 100% oxygen (0.5 l/min), and maintenance: 1.5% in 100% oxygen (0.5l/min)) on a heating pad (ATC2000, World Precision Instruments, Inc.). Hair and skin were removed from the dorsal surface of the skull, which was subsequently washed with saline and sterile cortex buffer. The headplate was then attached with dental cement (Super-Bond C&B; Sun Medical) to the bone posterior to bregma. Next, we made a craniotomy over VTA/SNc and injected 0.5 μl diluted viral construct (0.25 μl of AAV1.Syn.Flex.GCaMP6m.WPRE.SV40 diluted in 0.25 μl PBS) at ML: 0.5mm from midline, AP: -3 mm from bregma, DV: 4.4 mm from dura. An optic fiber (400 μm, NA: 0.48, Doric Lenses Inc.) was implanted over NAc (ML: 1 mm, AP: 1.25 mm, DV: -3.8 mm) in 4 mice (1 mouse was implanted in both left and right NAc, thus the data were collected from 5 brain hemispheres in total), and in the DMS (ML: 1 mm, AP: 1.25 mm, DV: -2.5 mm) in 5 mice (2 mice were implanted in both left and right DMS, thus DMS data were collected from 7 brain hemispheres in total). The fiber was also set in place with dental cement covering the rest of the exposed skull. For pain relief, Carprofen was provided in the cage water for 3 days after surgery (0.1 ml of 5% Carprofen mixed with 150 ml filtered tap water in the cage bottle). The implanted fibers did not substantially influenced decision making behavior of mice compared to animals without fiber implants performing the same task (*p*=0.43, Wilcoxon rank sum test). All experiments were conducted according to the UK Animals Scientific Procedures Act (1986) under appropriate project and personal licenses.

### Behavioral tasks

After 7 days of recovery from surgery, mice were placed on water control and following 3 days of handling and acclimatization, training began in the 2-alternative forced visual detection task (Burgess et al., 2017; Lak et al., 2020). Mice were trained using water as a reward. After the task, they received top-up fluids to achieve a minimum daily amount of 40 ml/kg/day. Body weight and potential signs of dehydration were monitored daily.

**Figure 1:**
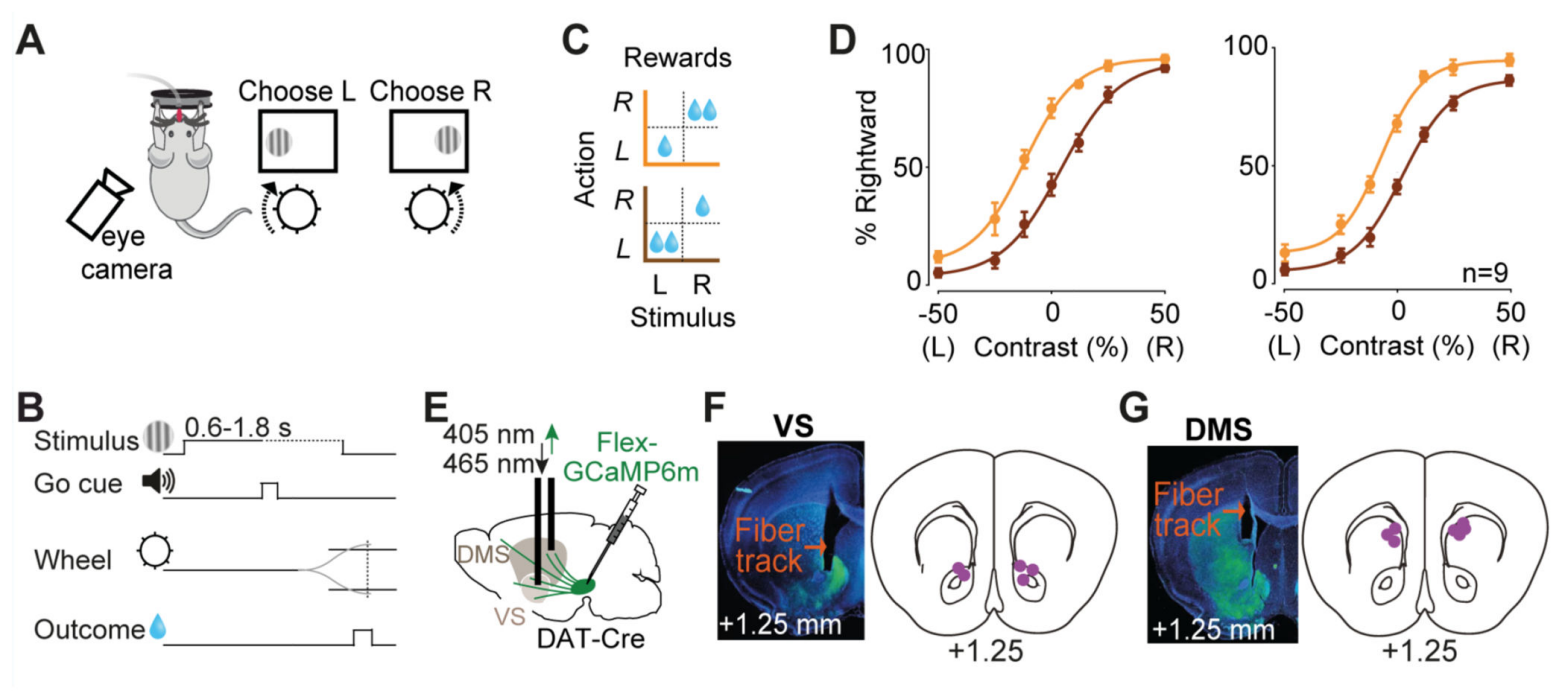
Imaging striatal dopamine axons during decisions requiring integration of sensory evidence and reward value. **A)** Task schematic. Mice were head-fixed in front of a screen displaying grating stimuli on the left or right side. Mice were rewarded with water for turning a steering wheel to bring the grating stimulus into the center. **B)** Task timeline. **C**) Reward size changed in blocks of 100-500 trials with larger reward available on either right (orange) or left (brown) correct choices. **D)** Left: Average psychometric curves of an example mouse (12 sessions), showing probability of choosing the stimulus on the right as a function of contrast on the left (L) or right (R), in the two asymmetric reward conditions (orange vs. brown). Right: population psychometric curves. **E)** Schematic of AAV-Flex-GCaMP6 injection into the midbrain of DAT-Cre mice and implantation of optic fiber above the ventral striatum (VS) or dorsomedial striatum (DMS). **F)** Left: Histological slide showing GCaMP expression (green) and position of optic fiber in the VS of an example animal. Right: Estimated position of fiber optic tips. **G)** The same as **F** but for DMS.

In each daily session, mice were head-fixed with their forepaws resting on a steering wheel (diameter: 62 mm). Trials began with an auditory tone (0.1 s, 12 kHz, ~40-50 dB) after the wheel was held still for at least 0.6 s (quiescence period). 0.7 s after the tone, a sinusoidal grating of varying contrast appeared on either the left or right side of the screen (19”, Iiyama, intensity measured in full black and full white: 1.3 and 201 Lux), positioned in front of the mouse (Fig. 1A,B). This was followed by a 0.6-1.8 s open loop period, during which mice could move the wheel but with no effect on the position of the grating. At the end of the open loop period, a distinct auditory tone marked the beginning of the closed loop period, during which mice were able to use the wheel to move the stimulus to the center of the screen to obtain a water reward. Water reward volume was either 1.4 μl or 2.4 μl depending on block and stimulus side (Fig. 1C). During training, parameters such as quiescence period, stimulus contrast, and open loop duration were gradually made more difficult. Within 2 weeks, mice had usually mastered the task, performing frequently above 85% (across all stimulus contrasts). In this task, the correct action to a stimulus on the left of the screen is to turn the wheel clockwise, which moves the stimulus from the left to center. We refer to this action as ‘contralateral’ action when recording from the right striatum (and vice versa for recordings in the left striatum).

Some mice (n=3) were additionally trained to perform a task variant that required refraining from wheel movements (Fig. 5). In this task, mice were trained to keep the wheel still prior to and after the stimulus onset, thus there was no wheel movement during correct trials. Following a 1 s quiescence period (i.e. no wheel movement), trials began with a grating stimulus appearing on the left or the right side of the screen. Mice were rewarded (2 μl water) for holding the wheel still for an additional 1.5 s. Wheel movement after the stimulus resulted in abortion of the trial and an auditory white noise.

The behavioral experiments were delivered by custom-made software written in Matlab (MathWorks) which is freely available (Bhagat et al., 2020). Instructions for both the software as well as hardware assembly are freely accessible at: www.ucl.ac.uk/cortexlab/tools/wheel.

#### Eye tracking

In 31 sessions we recorded 30 Hz video footage of the left eye. We used a camera (DMK 21BU04.H or DMK 23U618, The Imaging Source) with a zoom lens (ThorLabs MVL7000) focused on the left eye. To avoid contamination of the image by reflected monitor light relating to visual stimuli, the eye was illuminated with a focused infrared LED (SLS-0208A, Mightex; driven with LEDD1B, ThorLabs) and an infrared filter was used on the camera (FEL0750, ThorLabs; with adapters SM2A53, SM2A6, and SM1L03, ThorLabs). We acquired videos with MATLAB’s Image Acquisition Toolbox (MathWorks).

### Fiber photometry

Dopamine axon activity was measured using fiber photometry (Gunaydin et al., 2014; Lerner et al., 2015). We used multiple excitation wavelengths (465 and 405 nm) modulated at distinct carrier frequencies (214 and 530 Hz) to allow ratiometric measurements of calcium-dependent and calcium-independent (i.e. motion-related) changes in fluorescence. Light collection, filtering, and demodulation were performed as previously described (Lak et al., 2020) using Doric photometry setup and Doric Neuroscience Studio Software (Doric Lenses Inc.). For each behavioral session, least-squares linear fit was applied to the 405 nm isosbestic control signal, and the ΔF/F time series were then calculated as ((465 nm signal – fitted 405 nm signal) / fitted 405 nm signal).

### Histology and anatomical verifications

To verify the expression of viral constructs we performed histological examination. Mice were anesthetized and perfused, brains were fixed, and 60 μm coronal sections were collected. Confocal images from the sections were obtained using Zeiss 880 Airyscan microscope. We confirmed viral expression and fiber placement in all mice. The anatomical locations of implanted optical fibers were determined from the tip of the longest fiber track found, and matched with the corresponding Paxinos atlas slide (Fig. 1E-G).

### Statistical analyses

The presented analyses include 24,495 behavioral and neural trials (after the initial task learning was completed) recorded over a total of 87 sessions in 9 mice. The minimum and maximum number of trials per session were 103 and 640.

#### Normalization of neural activity

The neural responses collected in each session was first normalized by calculating z-scored ΔF/F. The data was further normalized by dividing the z-scored responses by the peak of averaged neural responses to stimuli with the highest contrast in each session. This ensured that the results when averaged across sessions or animals are not dominated by a small number of sessions or animals with stronger signals. We then averaged across all sessions of each animal before averaging the data across mice. These data were used for visualizing neural responses across time. For calculating neural responses in a specific time bin with respect to task events we used the normalized data as described above, and we subtracted the activity during a window before each event in each trial (-0.25-0 s) from the activity during a window (0.4-0.8 s) after the event in the same trial (Using 0.1-0.4 s post-event analysis window yielded comparable results in all our analysis). For animals with bilateral recordings, we first averaged the data across the two hemispheres (by grouping the data into ipsi- and contra-lateral with respect to each recorded hemisphere), before averaging the data across mice.

#### Pairwise comparisons and ANOVAs

We used neural responses measured in a specific time window after each task event (see above for the normalization and analysis time windows used). To test for statistical significance in the behavioral and neural data, we used standard statistical tests (Wilcoxon rank sum test or ANOVA across trials) as specified in each instance in the Results section.

#### Cross-validated regression analysis of neural data

**Figure 2:**
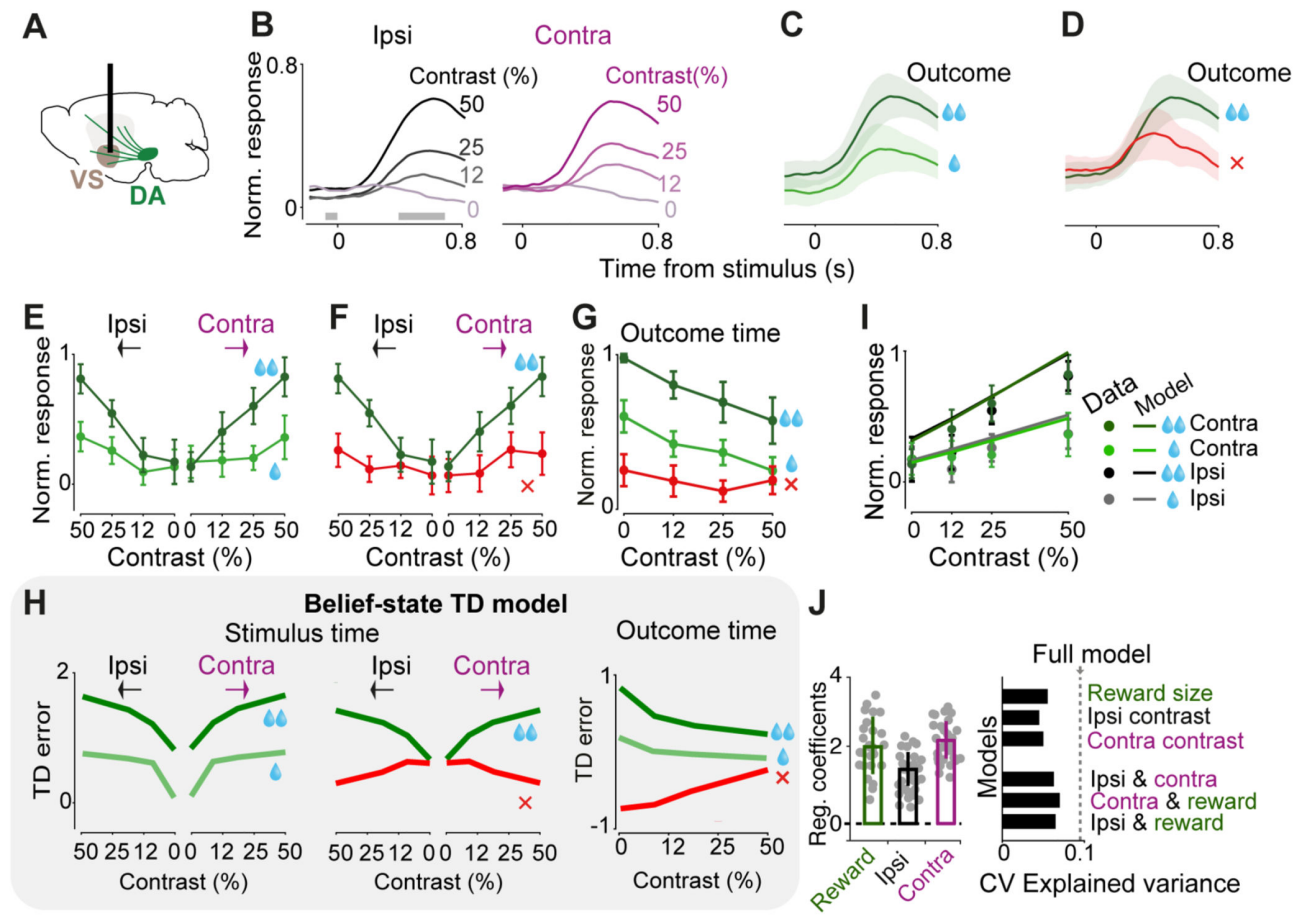
VS Dopamine axons respond to both contralateral and ipsilateral visual stimuli, and encode confidence-dependent prediction errors. **A)** Schematic showing imaging of VS dopamine axons. **B)** Normalized fluorescence following stimulus onset, separated by the contrast of grating stimulus presented ipsilaterally (left) or contralaterally (right). Fluorescence was normalized and averaged across mice (n=4, see Material and methods). Only correct trials that resulted in large reward are shown. Horizontal gray bars indicate the window used for the analyses in **E**, **F**. **C)** Same as **B**, for trials where a high-contrast (50%) contralateral stimulus was followed by correct choices leading to large (dark green) vs. small (light green) rewards. Shaded regions in this and subsequent figures show standard error of mean across mice. **D)** Same as **C**, for trials in which the choices were directed towards the larger-reward side correctly (dark green) or incorrectly (red). **E)** Average VS dopamine responses to stimuli as a function of stimulus contrast, separated by stimulus side and reward size. Responses reflect the difference in mean z-scored responses before and after stimulus onset (in the windows shown in **B**), normalized to the maximum response of each mouse, and then averaged across mice (see Material and methods). **F)** As in **E** but separated by trial outcome. **G**) Quantification of VS dopamine responses at the time of trial outcome (averaged across recordings from both hemispheres) separated based on the trial stimulus contrast and trial outcome. **H**) Schematic showing prediction errors of a temporal difference (TD) model that incorporates sensory decision confidence (i.e. subjective probability that the choice will be correct given the percept), adapted from Lak et al 2020. The TD errors at the time of stimuli and outcomes are scaled by the stimulus contrast, error/correct as well as the reward size, resembling VS dopamine responses shown in **E**-**G**. **I**) Lines are the fit of a regression model that includes contrast of both ipsi- and contralateral stimuli and reward size (see Material and methods). Circles are normalized responses to stimulus onset (averaged across mice). **J**) Left: average regression coefficients of the full model. Each dot is a session, and error bars are s.e.m across sessions. Right: Cross-validated regression analysis on stimulus responses. Dotted line indicates cross-validated proportion of explained variance by the full regression model. Top bars indicate explained variance of a reduced model consisting only of reward size, contrast of ipsi- or contralateral stimulus. Bottom bars indicate explained variance of reduced models each including two regressors. Hence the full model is necessary to account for the neural data.

In order to quantify the extent to which different trial features determined the magnitude of neural responses to stimuli in a trial-by-trial fashion, we modelled the changes in z-scored ΔF/F before and after stimulus onset (using temporal windows specified above) in a given trial *j,* which we denote as R*j*, as:

$$R_{j}={\text{β}}_{0}+{\text{β}}_{1}*{\text{c}}_{j}+{\text{β}}_{2}*{\text{i}}_{j}+{\text{β}}_{3}*{\text{i}}_{j}$$

where c*_j_* reflects contrast of contralateral stimulus, i*j* reflects the contrast of ipsilateral stimulus, and v*_j_* reflects the value of pending reward (0, 1.4, 2.4 for no reward, small reward and large reward). Z-scored stimulus contrast and reward sizes were used in the regression. β_1_, β_2_, and β_3_ are the coefficient weights for these variables, and β_0_ is an offset capturing mean fluorescence over all conditions. We tested reduced versions of the model omitting one or two terms out of [β_1_*c_*j*_], [β_2_*i*j*], and [β_3_*v_*j*_] to assess its performance compared to the full model. We used 5-fold cross validation (i.e. using 80% of trials to estimate regression coefficients and the remaining 20% of trials to compute explained variance) to estimate the explained variance of the model variants (averaged over sessions), and to select the best regression model for the neural data (Fig. 2J, 3J). Comparing the nested models using other model comparison methods such, as Akaike Information Criterion (AIC), revealed comparable results.

#### Eye movement analysis

Pupil location in 31 sessions was extracted from a 30 Hz video recording of the left eye using facemap (github.com/MouseLand/facemap) (Fig. 4A). Pupil location was defined as the centre of a 2D ellipse fitted to the pupil in each frame, and the trace was smoothed using a median filter (1 s window). 2D pupil location was projected along the single dimension of maximum variance (PCA), and then z-scored (Fig. 4B).

To assess the relationship between trial-by-trial DMS GCaMP fluorescence, stimulus contrast and pupil position, we used the following regression model:

$${\text{R}}_{j}={\text{β}}_{0}+{\text{β}}_{1}*{\text{c}}_{j}+{\text{β}}_{2}*{\text{i}}_{j}+{\text{β}}_{3}*{\text{P}}_{j}$$

Where R_j_ is the z-scored ΔF/F fluorescence averaged over a post-stimulus window (0.4-0.8 s) in trial *j*, p*_j_* is the pupil position averaged over the same post-stimulus window in trial j, c*_j_* denotes the contrast of contralateral stimulus and i*_j_* denotes the contrast of ipsilateral stimulus. Parameters (β_0_,β_1_,β_2_,β_3_) were fit by least-squares for each session separately. To illustrate the relationship between eye position and DMS dopamine signals (β_3_) after controlling for the confounding stimulus contrast (Fig. 4E), pupil position p was plotted against residual fluorescence R - (β_0_ + β_1_*c + β_2_*i). Using an analysis window of 0.1-0.4 s post-stimulus produced similar results.

## Results

### A decision task requiring integration of sensory evidence and reward value

We trained mice (n=9) in a two-alternative forced choice decision task that requires trial-by-trial evaluation of visual stimuli and reward values (Lak et al., 2020). Mice were head-fixed in front of a computer screen with their forepaws resting on a steering wheel. On each trial, a visual grating was displayed on either the left or right side of the screen at a variable contrast level, followed by an auditory Go cue presented after a 0.6-1.8 s delay (Fig. 1A,B). Mice were rewarded for turning the wheel after this cue, thereby bringing the grating into the center of the screen (Burgess et al., 2017). In trials with no stimulus on the screen (zero contrast), mice received rewards in 50% of trials. The volume of reward delivered for correct left and right choices was asymmetric, and the side giving larger reward was switched (without any warning) between blocks of 100-500 trials (Fig. 1C) (Lak et al., 2020). Mice learned to perform this task in 2-3 weeks of daily training. After the initial learning was completed, we collected 20,695 trials in 79 test sessions in 9 mice. Mice could detect high-contrast (easy) stimuli with an accuracy >90%, and low-contrast (difficult) stimuli near chance levels. Moreover, mice adjusted their choices to reward contingencies: the psychometric curves were shifted towards the side paired with larger reward (Fig. 1D) (Lak et al., 2020). The decisions were thus informed by both the strength of sensory evidence and the value of upcoming reward (contrast: *F*=256.5, *p*<0.000001, reward size: *F*=112.6, *p*<0.000001, ANOVA).

### Dopamine axons in ventral striatum respond to both contralateral and ipsilateral visual stimuli, and encode confidence-dependent prediction errors

While mice performed the task, we measured the activity of striatal dopamine axons using fiber photometry. We injected AAV containing Flex-GCaMP6m in the midbrain of DAT-Cre mice and implanted an optic fiber above ventral or dorsomedial striatum in different cohorts of mice (Fig. 1E-G).

The responses of VS dopamine axons to the visual stimuli scaled with expected reward size and with stimulus contrast, but showed no difference between ipsi- and contralateral stimuli (Fig. 2A-F). Following stimulus onset (i.e. prior to outcome onset, since a reward could only be received after the Go cue), VS dopamine responses were graded to the contrast of the stimulus, regardless of whether the visual stimulus appeared contralateral or ipsilateral to the recorded hemisphere (Fig. 2B; contrast: *F*=11.96, *p*<0.00001, ipsi/contra: *F*=0.39, *p*=0.53, ANOVA). The responses were also scaled to the size of upcoming reward (Fig. 2C, E; *F*=8.94, *p*=0.0053, ANOVA) and were larger in correct trials than in error trials (Fig. 2D, F; *F*=4.78, *p*=0.007, ANOVA). In order to statistically quantify the effects of contrast of ipsi- and contralateral stimuli and the value of pending outcomes on trial-by-trial responses of VS dopamine axons, we used regression models (see Material and methods). Specifically, we regressed time-binned neural responses against the contrast of ipsilateral stimulus, contrast of contralateral stimulus and the value of upcoming reward. This regression indicated that neural responses significantly encoded the contrast of both ipsi- and contralateral stimuli as well as upcoming reward value (Fig. 2 I,J left; *p*=0.0003, *p*=0.00001 and *p*=0.007 for ipsilateral stimulus, contralateral stimulus and upcoming reward, *F*=45.9, *p*<0.000001). We further confirmed these results using nested regressions that included one, two, or all regressors and used cross-validation to assess the predictive performance of each regressor (see Materials and methods). These regressions confirmed that the full model, i.e. the model that included contrast of both ipsi- and contralateral stimuli as well as upcoming reward value, accounts for the VS neural data better than models that include only one or two regressors (Fig. 2 J right).

Dopamine axons in VS appeared to encode neither the onset nor the direction of actions, i.e. the wheel movements for reporting choice. Action-locked signals in VS axons were present on average but absent in the subset of trials where the action was executed before the Go cue and therefore did not lead to reward (*p*=0.47, Wilcoxon rank sum test), suggesting that this activity is not actually related to movement. In these trials with early movement, stimulus-related responses were also attenuated, consistent with previous observations that VS dopamine release following a reward-predicting cue is attenuated unless a movement is correctly initiated (Syed et al., 2016).

The VS axons at the time of outcome strongly encoded the reward size (Fig. 2G) and the confidence in obtaining the reward, being largest when the reward was received in a difficult trial (Fig. 2G, *p*<0.05, Wilcoxon rank sum test between 0 versus 0.5 contrast for both small and large reward conditions).

These findings indicate that VS dopamine axons integrate reward value and sensory confidence. The VS dopamine signals at the times of both stimuli and outcomes resemble those we previously observed in VTA dopamine cell bodies during the same decision task (Lak et al., 2020). These responses resemble the prediction error term of a belief-state temporal difference (TD) reinforcement learning model that incorporates statistical decision confidence (i.e. subjective probability that the choice will turn out to be correct) into prediction error computation (compare Fig. 2E-G with Fig. 2H adapted from Lak et al., 2020). In such models, the difference between correct and error trials can arise before choice execution, and can be explained by the difference in statistical choice confidence (see Discussion).

### Dopamine axons in dorsomedial striatum respond to contralateral but not ipsilateral visual stimuli

**Figure 3:**
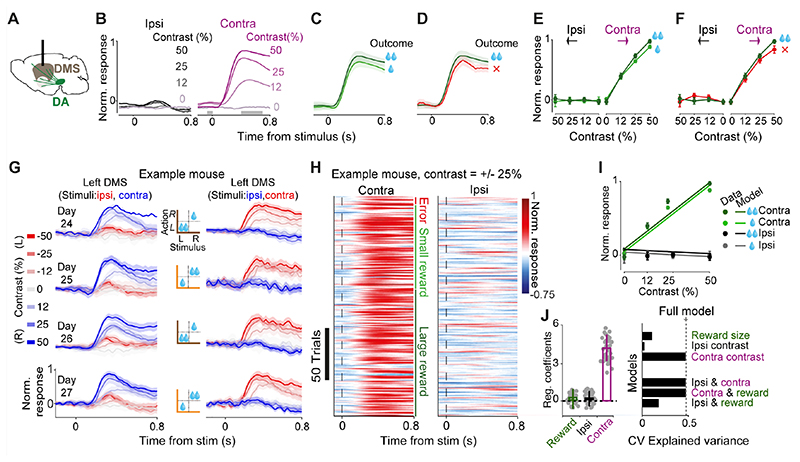
DMS Dopamine axons respond to contralateral but not ipsilateral visual stimuli. **A)** Schematic showing imaging of DMS dopamine axons. **B)** Normalized fluorescence following stimulus onset, separated by the contrast of grating stimuli presented ipsilaterally (left) or contralaterally (right). Fluorescence was normalized and averaged across mice (n=5). Only correct trials that resulted in large reward are shown. **C)** Same as **B**, for trials where a high-contrast (50%) contralateral stimulus was followed by correct choices leading to large (dark green) vs. small (light green) rewards. **D)** Same as **C**, for trials in which the choices were directed towards the large-reward side correctly (dark green) or incorrectly (red). **E)** Average DMS dopamine responses as a function of stimulus contrast, separated by stimulus side and reward size. Responses reflect the difference in mean z-scored responses before and after stimulus onset (in the windows shown in **B**), normalized to the maximum response of each mouse, and then averaged across mice. **F)** As in **E** but separated by trial outcome. **G)** DMS dopamine responses following stimulus onset recorded bilaterally in 4 consecutive sessions of an example mouse. Left column shows recordings in the left DMS, hence stimuli presented on the left and right side of the screen are ipsi- and contralateral respectively (and vice versa for recordings shown on the right column). Middle column shows reward contingency in each recorded session. Only rewarded trials are shown. Error bars are standard error of mean across trials. **H)** Trial-by-trial normalized responses in an example mouse for all trials in which the contrast of the stimulus was 25% either on the left or the right side. Trials are separated based on the trial outcome (error, small reward or large reward). **I)** Circles are normalized mean responses to stimulus onset, averaged across mice. Lines are predictions of the trial-by-trial regression model that only included contralateral stimulus contrast as a regressor (see Material and methods). **J**) Left: average regression coefficients of the full model, including the contrast of ipsi- and contralateral stimuli as well as the size of pending reward. Each dot is a session, and error bars are s.e.m across sessions. Right: Cross-validated regression analysis on stimulus responses. Dotted line indicates cross-validated explained variance by the full regression model. Top bars indicate explained variance of a reduced model consisting only of reward size, contrast of ipsi- or contralateral stimulus. Bottom bars indicate explained variance of reduced models each including two regressors. Hence, the model that only includes the contrast of the contralateral stimuli is sufficient to explain the neural data.

The stimulus-related activity of dopamine axons in the DMS differed from that in the VS in several ways (Fig. 3A-F, compare with Fig. 2A-F). First, dopamine axons in DMS responded to contralateral, but not ipsilateral, visual stimuli (Fig. 3B), and their responses scaled with the contrast of visual stimuli presented contralaterally (Fig. 3B, contralateral: *F*=243.3, *p*<0.00001, ipsilateral: *F*=0.12, *p*=0.94, ANOVA). Second, unlike the VS signals, dopamine responses in DMS were largely insensitive to the value of upcoming reward (Fig. 3C, E; *F*=0.93, *p*=0.18, ANOVA), and choice accuracy (Fig. 3D, F; *F*=3.4, *p*=0.09, ANOVA).

Lateralized responses to stimuli were evident in DMS dopamine signals from individual animals and in single trials (Fig. 3G,H). DMS dopamine axons recorded simultaneously bilaterally in individual animals responded strongly and rather exclusively to stimuli presented contralaterally: axons in the left and right hemispheres only responded to stimuli presented on the right and left side of the monitor respectively (Fig. 3G). Moreover, DMS dopamine axons showed robust responses to contralateral stimuli in individual trials of the task (Fig. 3H). In order to statistically quantify the effects of stimuli and outcomes on trial-by-trial responses of DMS dopamine axons, we used regression models identical to those used for analyzing VS dopamine signals (see Material and methods). The regression showed that neural responses encode the contrast of contralateral stimuli but not contrast of ipsilateral stimuli nor the value of pending reward (Fig. 3I,J left; *p*<0.000001, *p*=0.83 and *p*=0.59 for contralateral stimulus, ipsilateral stimulus, upcoming reward). Nested cross-validated regressions further confirmed these results, showing that the contralateral stimulus regressor is sufficient to match the explained variance of the full model (Fig. 3J right).

### DMS dopamine responses to contralateral stimuli cannot be explained by eye movements

**Figure 4:**
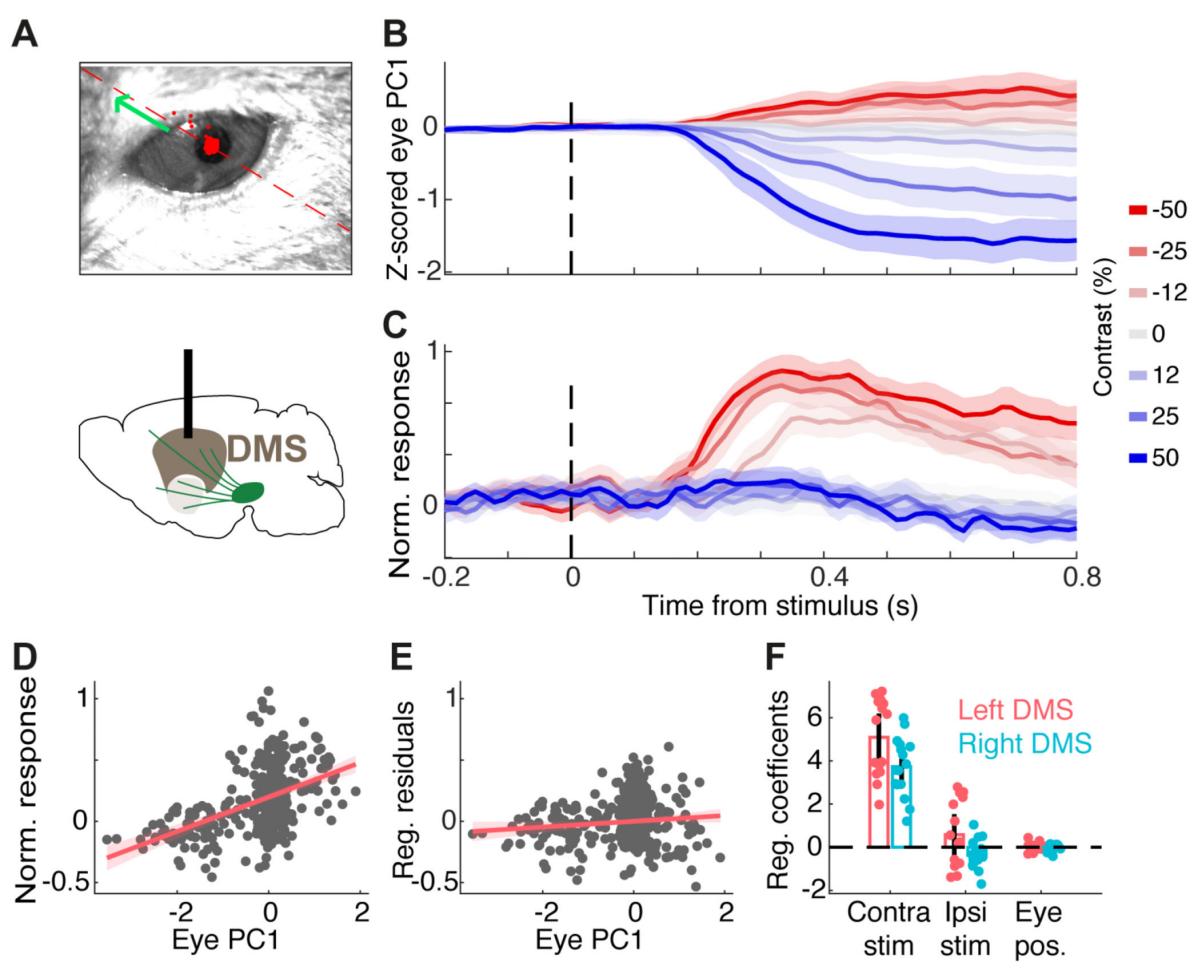
DMS dopamine responses to contralateral stimuli cannot be explained by eye movements. **A)** Example frame of the eye video. The red dashed line and green arrow indicates the positive direction of the 1^st^ principal component (PC) of 2D eye position. All sessions with eye recordings were of the left eye. **B)** Z-scored 1^st^ PC of pupil position in an example session. **C)** Dopamine signals recorded in the right DMS in the same session shown in B. **D)** The relationship between the 1^st^ PC of pupil position and neural signals in the example session, before adjusting for the effect of stimulus contrast. Each dot indicates one trial. **E)** The relationship between the 1^st^ PC of pupil position and neural signals after regressing out the confounding effect of stimulus contrast, indicating a negligible relationship between eye position and neural activity. **F)** The regression coefficients separately shown for sessions with left or right DMS dopamine recording in 5 mice. Each dot is one session and bars indicate averages across sessions. Coefficients of pupil position and ipsilateral stimuli were not significantly different from zero while coefficients of contralateral stimuli were significantly larger than zero (*p*=0.96, *p*=0.69, *p*<0.00001, respectively).

The responses of DMS dopamine axons to contralateral stimuli were not due to orienting movement such as eye movements (Fig. 4). While head-fixed mice cannot orient their heads towards the presented stimulus, we reasoned that they might rapidly move their eyes towards the stimulus presented on one side of the monitor and this could contribute to lateralized DMS dopamine responses. To assess this we extracted trial-by-trial pupil position from the recorded videos (Fig. 4A,B), and regressed DMS dopamine signals against eye position and contra/ipsi stimulus contrast (see Material and methods). After controlling for the stimulus contrast, the regression indicated that DMS dopamine signals were not significantly correlated with pupil movement (*p*=0.96). Rather, consistent with our previous analyses, these neural signals significantly reflected the contrast of contralateral visual stimuli (Fig. 4 C-F, *p*<0.00001). Thus, the responses of DMS dopamine axons reflect the contrast of contralateral stimuli, rather than orienting movements in responses to those stimuli.

DMS dopamine responses to contralateral stimuli are not due to task motor requirements

**Figure 5:**
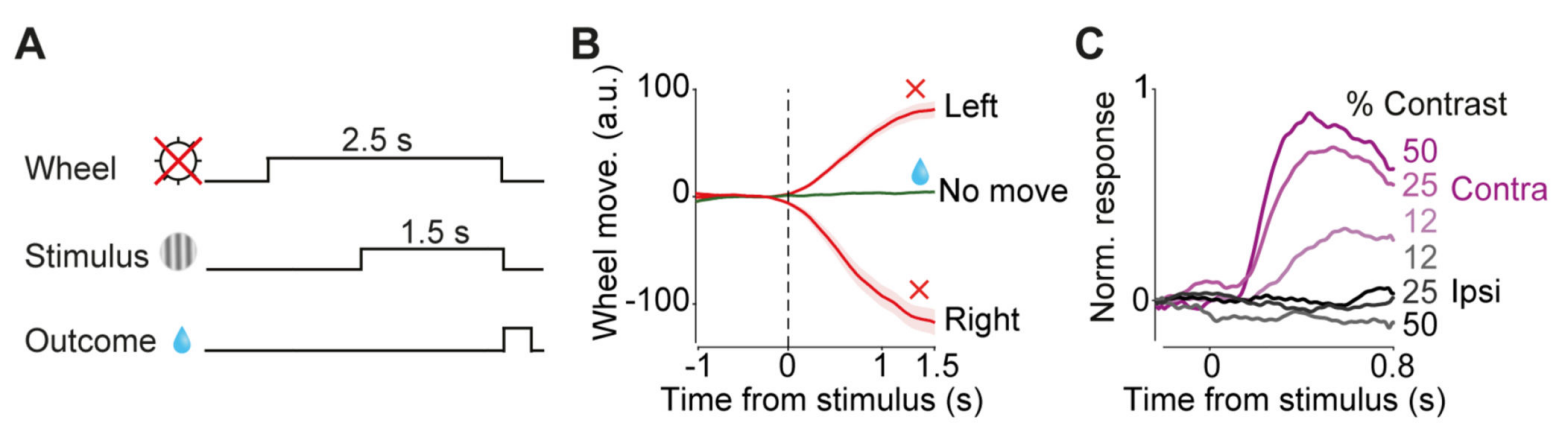
DMS dopamine responses to contralateral stimuli are not due to the task motor requirements. **A)** Schematic of no-movement task. After a 1.5 s period of no wheel movement, a stimulus appeared on the left or right side of the screen. Mice (n=3) had to hold the wheel still for a further 1.5 s to receive a reward. **B)** Wheel position in no-movement, move left, and move right trials averaged across all trials of all sessions. **C)** Stimulus aligned normalized mean DMS responses in trials in which mice successfully held the wheel still, separated by stimulus contrast.

Might the lateralized stimulus responses of DMS dopamine axons reflect some aspect of the upcoming planned movement, i.e. the directional wheel movements to report the choice? To test this, we measured DMS dopamine axon responses in a new ‘no-movement’ task. Mice were retrained to hold the wheel still for the whole trial: from 1 s prior to visual stimulus onset until 1.5 s after the visual stimulus, when they received reward (Fig. 5A,B). Wheel movement prior to the stimulus onset delayed the stimulus onset, and any wheel movement after stimulus onset aborted the trial (after an auditory white noise burst). After the initial training, we collected 3,800 trials in 8 test sessions in 3 mice. Mice learned to hold the wheel still in 40-60% of trials. We again observed strong responses of DMS dopamine axons in trials with contralateral visual stimuli and no wheel motion (Fig. 5C; ipsi vs contralateral: *F*=110.7, *p*=0.000001, contrast: *F*=16, *p*=0.00004, ANOVA). These results indicate that the contralateral visual responses of DMS dopamine axons are independent of the task’s motor requirements: they appear regardless of whether the stimulus instructs the animal to move or to refrain from moving.

### DMS dopamine axons encode specific combination of stimuli and actions in a lateralized manner

**Figure 6:**
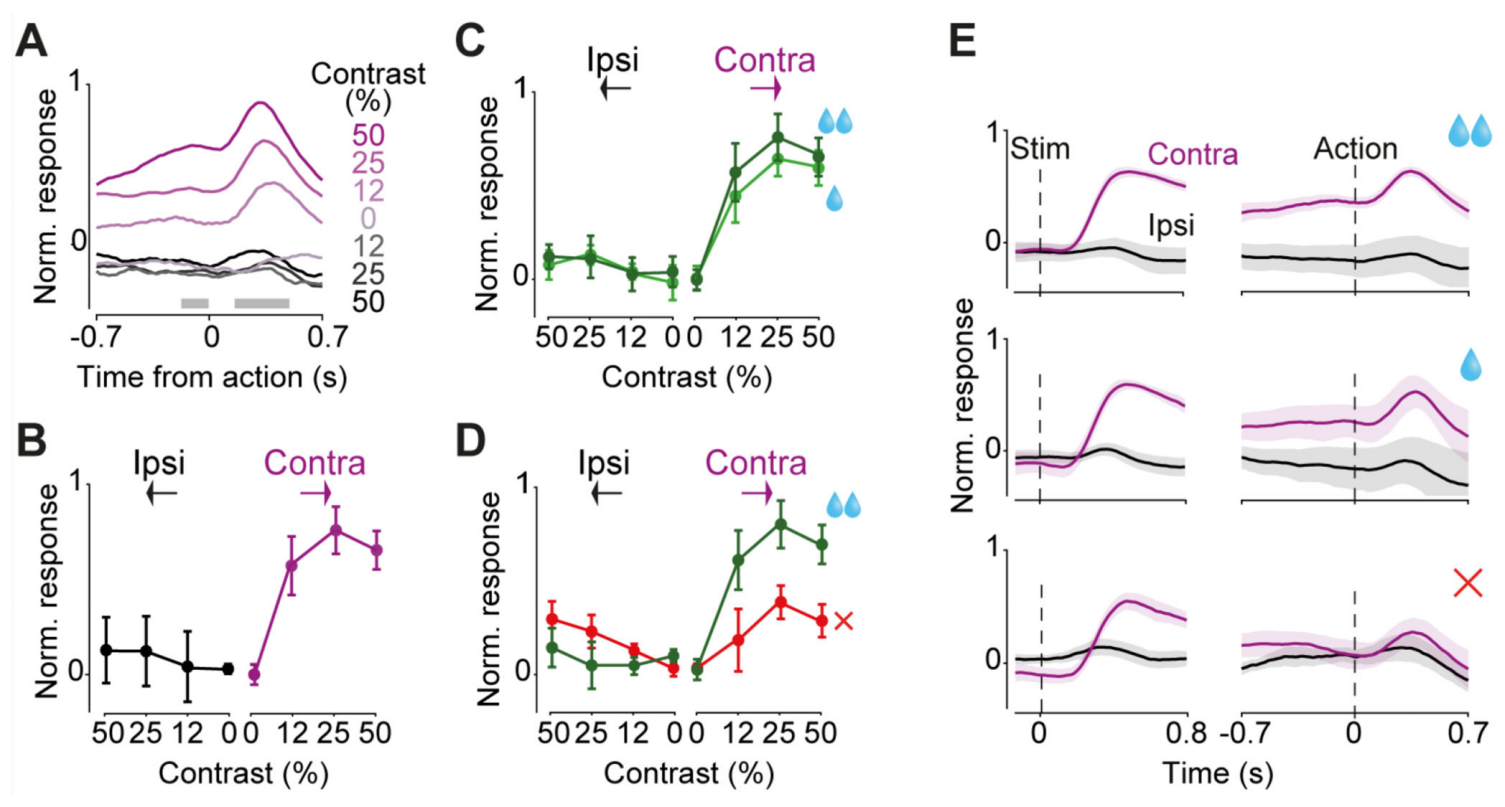
DMS dopamine axons encode specific combinations of stimuli and actions in a lateralized manner. **A)** Action-aligned signals during correct trials in DMS dopamine axons averaged across mice (n=5). Gray horizontal bars indicate the analysis window used in the subsequent panels. Note that the difference in responses prior to the action reflect responses to stimuli that preceded the action onset (see Fig. 3B). **B)** Average change in normalized neural responses after vs before action initiation. Responses reflect the difference in mean responses before and after the action onset (in the windows shown in A), normalized to the maximum response of each mouse, and then averaged across mice (see Material and methods). **C)** Average action-aligned signals separated by size of reward obtained. **D)** As in **C** but separated by choice accuracy. **E)** Summary of DMS dopamine signals during the choice task. Average stimulus responses of contralateral and ipsilateral DA axons in the choice task, separated by reward size and choice accuracy aligned to the stimulus onset (left) and action onset (right). Note that in the correct trials, contralateral action followed contralateral stimulus and in the error trials contralateral action followed ipsilateral stimulus. All panels show responses averaged across n=5 mice, and error bars are standard errors of mean across mice.

During the decision task (Fig. 1), dopamine activity in DMS was modulated not only at the onset of contralateral stimuli but also at the onset of actions, i.e. the onset of wheel movements leading to choice (Fig. 6). In this task the correct action to a stimulus on the left of the screen is to turn the wheel clockwise, which moves the stimulus from the left to center. We refer to this action as a ‘contralateral’ action when recording from the right striatum (and vice versa for recordings in the left striatum). DMS dopamine axons in the hemisphere contralateral to the stimulus showed robust responses to the contralateral action onset (Fig. 6A; *F*=7.99, *p*=0.0007, ANOVA) but not ipsilateral action onset (*F*=0.12, *p*=0.94, ANOVA). These signals occurred only when the visual stimulus was present (non-zero contrast trials) on the contralateral side but did not otherwise correlate with stimulus contrast (Fig. 6B; *F*=0.44, *p*=0.64, ANOVA), or with the size of upcoming reward (Fig. 6C; *F*=1.08, *p*=0.35, ANOVA). These contralateral action responses of DMS dopamine axons could not be explained by the movement of the visual stimulus on the screen, because it persisted in trials where mice responded before the auditory Go cue, and the visual stimulus did not yet move (*p*=0.021, Wilcoxon rank sum test). Nevertheless, the magnitude of DMS dopamine activity during contralateral actions was larger for correct than incorrect trials (Fig. 6D; *F*=12.41 *p*=0.0011, ANOVA). Thus, in addition to encoding contralateral visual stimuli, DMS dopamine axons encode correct (rewarded) contralateral actions, consistent with previous reports in freely moving mice (Parker et al., 2016). We did not observe prominent responses to rewards in the DMS dopamine axons in the decision task, consistent with past studies (Howe and Dombeck 2016).

Taken together, our results indicate that DMS dopamine axons encode a specific combination of stimuli and actions in a lateralized manner. Figure 6E, F summarize these. First, the DMS axons responded following contralateral stimuli but not ipsilateral stimuli (Fig. 6E, left). Second, these contralateral stimulus responses were followed by responses at the time of contralateral actions (Fig. 6E, right). Third, these contralateral action responses depended on choice accuracy, i.e. whether the ongoing choice is correct (Fig. 6E, right).

## Discussion

Our experiments reveal qualitatively distinct roles of dopamine circuitry across the striatum during visual decisions. Dopamine axons in dorsomedial striatum (DMS) responded to stimuli and actions in a strongly lateralized manner, signaling only contralateral stimuli (largely irrespective of the value of pending outcome) and rewarded, but not unrewarded (i.e. incorrect), contralateral actions. The contralateral DMS dopamine responses to stimuli could not be accounted for by eye movements towards stimuli, and persisted in a task variant with no movement, revealing the stimulus-related nature of these signals. For comparison, we also recorded dopamine axons in the ventral striatum (VS) which responded to stimuli and outcomes, encoding the confidence in receiving reward and the value of pending and received reward. These responses were largely independent of stimulus position on the screen and action direction.

Our results demonstrate that DMS dopamine axon activity encodes contralateral visual stimuli in behavioral tasks both with and without movement. Contralateral action responses of DMS axons have been reported previously (Parker et al., 2016; Tsutsui-Kimura et al., 2020), but our experiments using visual decision tasks extend these results in two ways. Firstly, lateralized DMS dopamine action signals depend on choice accuracy (i.e. for the same action they differ in error and correct trials), and secondly, DMS dopamine responses to visual stimuli are strongly lateralized. DMS dopamine responses to stimuli depended on the position and contrast of the stimulus and were evident regardless of whether the task required directional actions. Unlike in VS, the DMS dopamine responses prior to the outcome did not properly encode expected reward because they reflected stimulus contrast only unilaterally and had minimal encoding of reward size and choice accuracy.

The lateralized DMS dopamine signals we observed might shape various known features of dorsal striatal neuronal responses. Previous studies have identified prominent projections from visual cortical areas to the dorsal striatum (Hintiryan et al., 2016; Hunnicutt et al., 2016; Khibnik et al., 2014), and have shown that neurons in the dorsal striatum are particularly responsive to contralateral visual stimuli (Hikosaka et al., 1989; Peters et al., 2021). Given the role of dopamine signals in potentiating cortico-striatal synapses (Reynolds et al., 2001), their roles in rapid regulation of neuronal excitability in the striatum (Lahiri and Bevan, 2020), and evidence that striatal dopamine depletion alters striatal sensory responses (Ketzef et al., 2017), our results suggest that the lateralized dorsal striatal responses may be entrained by lateralized dopamine signals innervating this striatal region. Moreover, the graded response to stimulus contrast (which in our task determines the level of reward uncertainty) but limited encoding of pending reward value in the DMS dopamine axons might shape encoding of reward uncertainty observed in dorsal striatal neuronal responses (White and Monosov, 2016).

Our results help clarify the sensory vs action roles of dorsal striatal dopamine in visually-guided behavior. An early set of studies lesioned dorsal striatum dopamine unilaterally in a task in which freely-moving rats had to make a left or right movement to report the position of a flash of light. These studies concluded that the lesion-induced behavioral deficits (slow and impaired response to contralateral stimuli) were due to impairment in initiation of contralateral actions rather than a deficit in localizing the contralateral stimulus (Brown and Robbins, 1989; Carli et al., 1985). Later studies using single-unit recording in primates or calcium imaging in mice show that some dopamine neurons show stronger responses to contralateral, compared to ipsilateral visual stimuli (Engelhard et al., 2019; Kawagoe et al., 2004; Kim et al., 2015). Among these, by recording single putative dopamine neurons in primates, Kim et al 2015 extensively studied these neural responses in simple visually-guided saccade tasks, and demonstrated that a subgroup of dopamine neurons located in the lateral substantia nigra and projecting to the caudate have stronger responses to contralateral visual stimuli, and respond to visual stimuli with little dependence on the reward value of the stimulus. These more recent studies therefore identify a strong sensory component in dopamine responses, akin to the DMS dopamine axon responses we observed in our visual decision task in mice. Further studies will be required to establish the precise causal impact of these signals in visual decisions.

Our results also reveal the encoding of confidence-dependent reward prediction errors in the mesolimbic dopamine pathway. The responses of dopamine axons in VS at the time of stimuli and trial outcome scale with the sensory evidence, choice accuracy as well as reward value, resembling prediction error term of a belief-state reinforcement learning model that

incorporates statistical decision confidence (estimated, for instance, using signal detection theory) into prediction error estimation (Lak et al., 2020). These VS dopamine signals are similar to the responses of dopamine cells bodies in the VTA imaged in the same task in mice (Lak et al., 2020), and of spiking activity of putative individual dopamine neurons recorded in a similar task in primates (Lak et al., 2017) which also encoded prediction errors scaled to the statistical confidence in obtaining the reward as well as reward value. In both VS dopamine axon signals, as well as in our previous recordings from dopamine cell bodies (Lak et al., 2017; Lak et al., 2020), the difference between correct and error trials emerged prior to the trial outcome. These early differences could be accounted for by the belief-state reinforcement learning model because in such models the choice confidence can be estimated prior to the choice execution, and it is lower in the error trials compared to correct trials. Thus, the VTA confidence-dependent dopamine signals appear to be carried forward to ventral regions of striatum. On the other hand, the lateralized DMS dopamine signals to stimuli and actions cannot be explained by canonical prediction error framework, as has been shown previously in the case of the action signals (Howard et al., 2017; Lee et al., 2019; Tsutsui-Kimura et al., 2020).

Our findings are consistent with the idea that dopamine projections to dorsal striatum promote the association between contralateral stimuli and contralateral actions, whereas projections to ventral striatum promote the association between stimuli and outcomes. Dorsal striatum is necessary for executing lateralized goal-directed actions and for maintaining stimulus-action associations (Balleine et al., 2007; Brasted et al., 1997; Featherstone and McDonald, 2005; Jog et al., 1999; Miklyaeva et al., 1994; Tai et al., 2012; Yin et al., 2005). During sensory decision making, manipulation of cortico-striatal neurons, terminating in the dorsal striatum, biases choices in 2-alternative sensory decision task (Znamenskiy and Zador, 2013). Moreover, the strength of cortico-striatal synapses increases in a stimulus-selective manner as animals learn to perform a sensory decision task (Xiong et al., 2015). These synapses are under heavy influence of dopamine. Accordingly, the DMS

dopamine responses to contralateral stimuli and contralateral rewarded actions we observed here might contribute to forming associations between specific stimuli and actions. Our results on dopamine axons in the ventral striatum are consistent with the role of this striatal region as well as the role of dopamine in this region in forming stimulus-outcome associations (Robbins and Everitt, 1992; Rothenhoefer et al., 2017). Thus, anatomically-organized dopamine modulation of striatum can support distinct associations between stimuli, actions and outcomes, thereby refining goal-directed decisions.

## Significance statement

While the striatum is central to goal-directed behavior, the precise roles of its rich dopaminergic innervation in perceptual decision-making are poorly understood. We found that in a visual decision task, dopamine axons in the dorsomedial striatum (DMS) signaled stimuli presented contralaterally to the recorded hemisphere, as well as the onset of rewarded actions. Stimulus-evoked signals persisted in a no-movement task variant. We distinguish the patterns of these signals from those in the ventral striatum. Our results contribute to the characterization of region-specific dopaminergic signaling in the striatum and highlight a role in stimulus-action association learning.
